# Cell culture-derived influenza vaccines in the severe 2017–2018 epidemic season: a step towards improved influenza vaccine effectiveness

**DOI:** 10.1038/s41541-018-0079-z

**Published:** 2018-10-09

**Authors:** Ian G. Barr, Ruben O. Donis, Jacqueline M. Katz, John W. McCauley, Takato Odagiri, Heidi Trusheim, Theodore F. Tsai, David E. Wentworth

**Affiliations:** 1 WHO Collaborating Centre for Reference and Research on Influenza, The Peter Doherty Institute For Infection And Immunity, 792 Elizabeth Street, Melbourne, 3000 Australia; 2 Biomedical Advanced Research and Development Authority, Influenza and Emerging Infectious Diseases Division, 300 Independence Avenue, SW, Washington, DC 20201 USA; 3 Influenza Division, Centers for Disease Control and Prevention (CDC), 1600 Clifton Road MS A-20, Atlanta, GA 30329-4027 USA; 4 WHO Collaborating Centre for Reference and Research on Influenza, Crick Worldwide Influenza Centre, The Francis Crick Institute, 1, Midland Road, London, NW1 1AT UK; 5 WHO Collaborating Centre for Reference and Research on Influenza, National Institute of Infectious Diseases, Influenza Virus Research Center, 4-7-1 Gakuen, Musashi-Murayama-shi, Tokyo 208-0011 Japan; 6 IDT Biologika GmbH, Am Pharmapark, 06861 Dessau-Rosslau, Germany; 7 Takeda Vaccines (USA), 75 Sidney St, Cambridge, MA 02139 USA; 8 Influenza Division, Centers for Disease Control and Prevention (CDC), 1600 Clifton Road MS A-20, Atlanta, GA 30329-4027 USA

## Abstract

The 2017–2018 seasonal influenza epidemics were severe in the US and Australia where the A(H3N2) subtype viruses predominated. Although circulating A(H3N2) viruses did not differ antigenically from that recommended by the WHO for vaccine production, overall interim vaccine effectiveness estimates were below historic averages (33%) for A(H3N2) viruses. The majority (US) or all (Australian) vaccine doses contained multiple amino-acid changes in the hemagglutinin protein, resulting from the necessary adaptation of the virus to embryonated hen’s eggs used for most vaccine manufacturing. Previous reports have suggested a potential negative impact of egg-driven substitutions on vaccine performance. With BARDA support, two vaccines licensed in the US are produced in cell culture: recombinant influenza vaccine (RIV, Flublok™) manufactured in insect cells and inactivated mammalian cell-grown vaccine (ccIIV, Flucelvax™). Quadrivalent ccIIV (ccIIV4) vaccine for the 2017–2018 influenza season was produced using an A(H3N2) seed virus propagated exclusively in cell culture and therefore lacking egg adaptative changes. Sufficient ccIIV doses were distributed (but not RIV doses) to enable preliminary estimates of its higher effectiveness relative to the traditional egg-based vaccines, with study details pending. The increased availability of comparative product-specific vaccine effectiveness estimates for cell-based and egg-based vaccines may provide critical clues to inform vaccine product improvements moving forward.

## The 2017–2018 influenza epidemic revived public health concerns

The 2017–2018 influenza season in the United States was in many respects the most severe since the A(H1N1) pandemic of 2009. Influenza virus circulation began to increase in early November and then increased rapidly from December through early February. A(H3N2) subtype viruses predominated over influenza B and A(H1N1), until late in the season when influenza B virus activity peaked. The levels of influenza-like illness, which reflects outpatient visits and emergency department visits, were the highest seen in recent influenza seasons including those when A(H3N2) predominated.^1^ In contrast to previous years, the US 2017–2018 season was characterized by widespread and high intensity virus circulation at the same time in most states^2^ Cumulative rates of influenza-confirmed hospitalizations exceeded those seen in the 2014–2015 season, an A(H3N2) predominant season categorized as one of high severity.^3^ In Europe, A(H3N2) viruses also co-circulated in many countries along with A(H1N1) and influenza B viruses, while in the Southern Hemisphere season in Australia A(H3N2) viruses and to a lesser extent B/Yamagata viruses predominated during the biggest influenza season in the last 20 years.^4–6^

Seasonal epidemics dominated by A(H3N2) typically result in higher rates of infection, hospitalization, and death, especially among the elderly compared with outbreaks due to other subtypes. Based on estimates of influenza disease burden, in A(H3N2) epidemics like those in the 2012–2013 and 2014–2015 seasons, Centers for Disease Control and Prevention (CDC) predicted that as many as 35.6 million illnesses, 16.6 million medically attended visits, 710,000 hospitalizations, and 56,000 deaths could result in such seasons.^7^ Similarly, excess winter mortality in Europe was notably elevated in winter epidemics when influenza A(H3N2) viruses predominated.^8^

Vaccination remains the best available intervention to prevent morbidity and mortality caused by the four different influenza A and B viruses currently co-circulating in humans. The interim vaccine effectiveness (VE)^9^ against A(H3N2) during the 2017–2018 season was lower than prior multi-year pooled VE estimates^10^ in mid-season reports from the Northern Hemisphere, including adults 19–64 years of age from the United States,^11^ Canada,^12^ and Europe,^13^ with similar observations from Australia for the 2017 epidemic season in the Southern Hemisphere.^14^ Low VE is typically associated with substantial antigenic differences between the vaccine virus and circulating viruses that result from the rapid evolution of co-circulating variants that are selected due to escape from host immunity, as was observed in the 2014–2015 influenza season.^15^ However, although the co-circulating A(H3N2) subtype viruses that predominated in the 2017–2018 season comprised many HA genetic clades, no significant antigenic drift from the A/Hong Kong/4801/2014-like cell-propagated reference virus was detected,^16^ indicating an optimal vaccine strain selection. Nevertheless, 36% of A(H3N2) viruses co-circulating in the US from October to early February, showed reduced inhibition by antisera raised to egg-adapted viruses used for production of influenza vaccines in embryonated hen’s eggs.^17^ A high proportion of A(H3N2) viruses from Europe were also antigenically distinct from the egg-propagated vaccine reference virus.^5^ Furthermore, only a small proportion of A(H3N2) isolates could be characterized by hemagglutination inhibition tests and had to be analyzed by other methods such as virus neutralization. One of the multiple factors implicated in reduced VE in 2017–2018 are the changes acquired in the viral hemagglutinin (HA), upon isolation, adaptation, and propagation in eggs, which are the base of the manufacturing system currently used globally^18^ (Table 1).

**Table 1:** Comparative characteristics of selected seasonal influenza vaccines^a^

	**Egg-based inactivated virus vaccine**	**Cell-based inactivated virus vaccine**	**Recombinant HA vaccine**
Immunogen production	Influenza virions produced in eggs or cell cultures are purified, lysed with detergent to release hemagglutinin (HA) and neuraminidase (NA) oligomers, which form “rosettes”		Insect cells are lysed with detergent to release HA oligomers, which form “rosettes”. Does not contain NA
Required seeds	Candidate vaccine virus (CVV) “seed” must be produced – typically several weeks; possibly very few suitable CVV’s become available	Candidate vaccine virus (CVV) “seed” must be produced – typically several weeks; generally several suitable CVV’s become available	Recombinant vaccine virus “seed” must be produced – typically several weeks; do not need CVV, just HA sequence
Mutation risk	Propagation of CVV in eggs selects mutations that decrease antigenic relatedness to native virus and may impact vaccine effectiveness	Production of CVV in mammalian cells minimizes risk of mutation and potential impact on vaccine effectiveness	Product made from stable (cell isolate) gene sequence, negligible mutation risk, but glycosylation may vary depending on host cells
Immunogen yields	Variable depending on virus strain – often improved by further passaging or reassorting CVV (with increased risk of further mutations)	Variable depending on virus strain – may be improved by further passaging or reassorting CVV	Consistent productivity independent of virus strain, additional optimization of process possible
Vaccine manufacture cost	Low – eggs are a relatively inexpensive production platform	Greater than egg-based – improvement may be possible with process optimization and larger production scale	
Current US-licensed manufacturers	GSK Sanofi Pasteur Seqirus	Seqirus	Protein Sciences Corp. (now Sanofi Pasteur)
Current share of US market	85–90%	10–15%	1–2%

^a^Based on influenza vaccines licensed for use in the United States

Influenza vaccines manufactured in eggs for the 2017–2018 season used a seed virus with three amino-acid substitutions in the HA (N96S, L194P, and T160K, which resulted in the loss of a glycosylation at residue 158) that occurred when viruses from humans were isolated and propagated in eggs and one additional substitution at position 225 (Asp to Gly) after virus reassortment in the laboratory, a requirement for large-scale vaccine manufacturing. Although many other egg-adapted viruses were isolated and tested by WHO-CCs they had other HA mutations that dramatically impacted the antigenic properties and these were not taken forward. Several studies have hypothesized that certain egg-adaptive mutations appear to change viral antigenicity and therefore could be partially responsible for the lower than expected VE.^12,19^ The occurrence of changes to the HA of human influenza viruses propagated in eggs was recognized decades ago but the detrimental impact of these mutations on vaccine antigenicity has largely had been averted by the WHO Collaborating Centers for influenza (WHO-CC) through the identification of optimal candidate vaccine viruses (CVVs) from the available egg-adapted strains.^20–22^ However, the evolution of A(H3N2) subtype viruses in recent years has resulted in viruses that limit the availability of optimal egg-based vaccine strains. This challenge has added further impetus to the expanded utilization of alternative host systems for vaccine manufacturing (cell-based or recombinant protein-based vaccines), which were concurrently being launched to strengthen public health preparedness for seasonal and pandemic influenza emergencies^23,24^ (Table 1).

## Cell-based and recombinant influenza vaccines

Flucelvax™, was initially licensed as an inactivated trivalent cell culture-grown vaccine (ccIIV3) (originally manufactured using egg-derived vaccine seed viruses) propagated in a qualified mammalian cell line (proprietary 33016PF Madin-Darby Canine Kidney (MDCK)) and was licensed in 2012 by Novartis Vaccines and Diagnostics, Inc. (in 2015, bioCSL acquired Novartis’ influenza vaccine assets and created Seqirus, based in London, UK). This trivalent vaccine was upgraded to a quadrivalent vaccine (ccIIV4 (Flucelvax™)) that was licensed by the Food and Drug Administration (FDA) in 2016. However, both these vaccines were produced from egg-derived virus seeds and therefore, mutations associated with adaptation to eggs were likely still present in the vaccine antigen and effectiveness was likely to be similar to a licensed egg-based vaccine.^25^ In August 2016, Seqirus received FDA supplemental approval for the use of CVVs that had been isolated and propagated in MDCK cells at either of two supporting WHO-CCs (Centers of Disease Control and Prevention in the US and the Victorian Infectious Diseases Reference Laboratory in Australia) for the manufacture of ccIIV4.^26–28^ This enabled the production of completely cell-derived A(H3N2) influenza virus from the initial virus isolation through to the full manufacture of the vaccine. Thus, in the Fall of 2017, for the first time, a seasonal influenza vaccine (Flucelvax Quadrivalent™) was administered to millions of people in the US, which contained a purely mammalian cell culture-derived influenza A(H3N2) virus as one of its four components.^26^ Preliminary estimates of relative effectiveness conducted by the FDA based on health insurance claims data from the Centers for Medicare Services indicate that ccIIV4 provided a modest improvement over egg-based vaccines in subjects 65 years of age and older.^18,29^

The use of a completely cell culture-derived virus in a vaccine, which has not been propagated at any stage in eggs, was the fruition of a 10-year effort that began in 2007 (the key personnel involved in this program are listed in the Acknowledgements). The then Novartis Vaccines and Diagnostics (now Seqirus) joined forces with the WHO-CCs in the US (CDC, Atlanta), the Melbourne-based WHO-CC, and subsequently, with the WHO-CCs in London and Tokyo through several research agreements supported in part by the US Department of Health and Human Services (HHS).^30^ Under these agreements, the Novartis’ proprietary 33016PF MDCK cell line, which had been qualified and used for vaccine manufacturing, was transferred to the respective WHO-CCs, along with specified media and standard operating procedures to enable isolation of influenza viruses in these cells directly from primary patient samples, for further propagation, characterization and eventual disbursement to cell culture-based vaccine manufacturers.^31^

These early studies showed that the influenza A(H3N2) virus isolation rate in the 33016PF cells was orders of magnitude greater as compared with eggs, while at the same time avoiding the egg adaptation changes of the human influenza viruses that occurs when these viruses are propagated in eggs.^32–34^ The extent to which seasonal vaccines that are fully derived and manufactured in mammalian cell culture will provide improved VE over egg-manufactured influenza vaccines remains to be determined, as definitive comparative field trials have not been reported yet, but animal and human serological testing suggests that mammalian cell-derived antigen could be, at the least, more protective – in certain seasons – particularly with respect to A(H3N2) and B vaccine components.^22,35–37^

In 2013, the FDA-approved Flublok™ (Protein Sciences Corp., recently acquired by Sanofi Pasteur, based in Lyon, France), which is a vaccine composed of only influenza recombinant HA (recombinant influenza vaccine, RIV) that is manufactured using baculovirus overexpression and purification from infected insect cell cultures (*Spodoptera frugiperda*, fall army worm). RIV is designed to contain the amino-acid sequence of the HA ectodomain of the cell culture-propagated vaccine prototype viruses that are recommended by WHO. In addition, the RIV manufacturing does not include chemical virus inactivation treatment, which may also alter the vaccines’ antigenicity^38–40^ and contains increased concentration of HA protein (threefold) as compared with IIV. In a randomized controlled trial in adults 50 years or older comparing RIV4 with a licensed egg-grown quadrivalent vaccine, RIV4 was 36% more efficacious (95% confidence interval (CI): 14–53%) in preventing RT-PCR confirmed influenza A and 30% more efficacious overall; 80% of the influenza A viruses identified in this 2014–2015 trial were H3N2 strains.^41^ Unfortunately, it is difficult to differentiate the respective contributions of the vaccine’s higher HA antigen content and/or lack of egg-adaptive changes towards the higher relative efficacy.

Considering the preliminary level of VE evidence and the limitations of observational studies,^42^ it will be critical to conduct larger and more rigorous trials as soon as possible. Opportunities for future measurements of RIV4 VE are limited by production and distribution levels in the US, which are not high enough to assess its performance through the established effectiveness monitoring platforms.^10,43^ Even a modest 5–10 percentage point increase in VE would have prevented an additional 494,000–982,000 illnesses and 11,500–22,800 hospitalizations in the 2016–2017 US influenza season.^44^ Although further studies are needed to evaluate more fully the potential benefits of cell-based vaccine in reducing the burden of influenza disease, in particular due to A(H3N2) viruses, more transformative product development will ultimately be needed to achieve optimal effectiveness of influenza vaccines overall. The interim VE gains achieved through cell-based and/or recombinant manufacturing platforms could be further leveraged in many directions, including: (i) enhancing immune responses and protective efficacy by formulation with adjuvants such as MF59®, as demonstrated by studies with FluAd^45^; (ii) enhancing immunogenicity and effectiveness by higher antigen dose formulations as shown by studies with Fluzone™ High Dose^46,47^; (iii) the potential for producing vaccine antigen in tobacco plants (which are awaiting results from pivotal clinical studies)^48^; (iv) formulating vaccines in which only one [i.e., A(H3N2)] of the three or four components is produced using cell-based or recombinant systems, while others are still produced in eggs.

## Monitoring product-specific vaccine effectivess

Observational influenza VE studies are crucial for assessing vaccine performance in “the real world”, informing on vaccine regulation, policy, and product development. However, current data streams supporting observational influenza VE studies do not yield vaccine product-specific information to assess the relative effectiveness of each of the 11 vaccines distributed in the United States in 2017–2018. Although the use of RIV and ccIIV may offer the potential for better protection over traditional egg-based influenza vaccines, sparse utilization of these vaccines to date does not allow robust comparative effectiveness estimates using current observational studies to support preferential use of one injectable influenza vaccine over another.^41^ The clinical information gap on the relative effectiveness in the current season has become the focus of intense activity. The FDA has recently announced preliminary results of a study to assess relative effectiveness of ccIIV and egg-based vaccines in persons 65 years and older by examining a US national database of insured benefit claims.^18,29^ Two additional ongoing studies sponsored by BARDA and the US Department of Defense are expected to provide absolute and relative effectiveness data for ccIIV and egg-based vaccines in subjects between the ages of 4 and 64 years or 4 years and above, respectively.

Development of improved seasonal influenza vaccines will depend on innovative clinical studies evaluating differences between product-specific or platform-specific VE to generate evidence to inform manufacturers, regulators, public health policy makers, and consumers. As the list of new vaccines grows, we need to reassess the best methods to monitor VE of new products and to compare products, including expanding current clinical studies and networks, harmonizing data collection to allow combined individual data analyses and meta-analyses, and designing prospective randomized designs to provide a robust foundation to support short- and long-term efforts to reduce the public health impact of seasonal and pandemic influenza.

## The role of private–public partnerships

The Pandemic and All-Hazards Preparedness Act promulgated in 2006^49^ authorized the US Department of Health and Human Services (HHS) to support flexible public–private partnerships (PPPs) to achieve pandemic preparedness goals through development of innovative medical products, including vaccines, drugs, respirators, and diagnostics. Seasonal and pandemic influenza prevention and response are inextricably linked, as preparedness for seasonal influenza viruses is the foundation for preparedness for an influenza pandemic. Starting in 2006, HHS awarded several contracts to companies to establish influenza vaccine manufacturing capacity to support pandemic vaccination response capability. Two major thrusts of the program were focused on development of cell-based and recombinant protein-based vaccines by creating the necessary infrastructure.

In 2007, Novartis V&D and the WHO-CC at the CDC signed a Cooperative Research and Development Agreement to support the use of viruses isolated using cell culture to produce the cell-based influenza vaccine. Similar agreements were established between Novartis and the WHO-CCs in London, Melbourne, and Tokyo. These collaborations were aimed at establishing laboratory protocols and workflows to optimize isolation of viruses suitable for development of seed stocks to manufacture vaccines under a quality system in compliance with cGMP FDA guidelines. Since the inception of the collaborative agreements, the WHO-CCs in Atlanta and Melbourne, have isolated and characterized hundreds of viruses to generate the evidence base required to support the recent regulatory approval by the FDA. A range of MDCK and other manufacturing cell lines were tested to identify a single line that was best suited for the primary isolation of influenza viruses from clinical specimens while excluding adventitious agents, preserving genetic and antigenic characteristics of the original isolate, and allowing for sufficient manufacturing yield in cells and eggs.^32–34^ The 33016PF MDCK cell line developed by Novartis Vaccines and Diagnostics was identified as the best suited among the lines that were evaluated. Although the 33016PF MDCK cell line is being used at the CDC and Melbourne WHO-CCs, the Tokyo CC developed its own qualified MDCK cell line to provide CVVs for manufacturers.^50^ Such collaborations are the most recent examples of the longstanding PPPs, which are necessary to meet the stringent bi-annual timelines for seasonal influenza vaccine campaigns and now have resulted in the licensure of new non-egg-based vaccines. Cell-based and recombinant protein vaccine technologies offer greater flexibility to realize the potential for more effective vaccines than those produced in eggs while possibly supporting accelerated timelines for vaccine composition updates to keep pace with rapid evolution of influenza viruses or to respond to the inevitable future pandemic(s). The PPPs are excellent examples to illustrate the HHS strategy to establish sustainable pandemic preparedness while promoting seasonal vaccine improvement. Despite the foregoing desirable features of cell-based and recombinant vaccines, during the 2017–2018 influenza season these vaccine technologies only represented approximately 13% (ccIIV) and 1% (RIV), respectively, of all vaccine doses distributed in the US^43^ and broader implementation of these and other new-generation vaccines may not occur until there is a consistent body of evidence supporting their improved VE.

The severity of the 2017–2018 influenza season in North America and elsewhere was greater than expected, even when the predominance of an A(H3N2) subtype was taken into account. Innovative vaccines that have recently become available represent the culmination of decade-long efforts by industry and public partners to diversify influenza vaccine manufacturing platforms. The new egg-independent platforms represent a milestone that is now being realized, as these vaccines have now been given to millions of people in the US.^26,41,51–53^ The culmination of this work for ccIIV4 will be materialized when all four components of the seasonal influenza vaccines are derived from production systems that yield optimal immunogens without host selected mutations. The higher relative efficacy of RIV during 2014–2015, principally on the H3N2 subtype, is encouraging, and additional observations on its relative effectiveness against other viral subtypes and during additional seasons, would be welcome. As each of these practical steps is taken, it is anticipated that there will be a subsequent rise in the effectiveness of seasonal influenza vaccines. However, additional work to understand host-related immune factors such as the impact of prior exposure and immunity to a subsequent vaccine response will also likely lead to strategies that improve influenza VE. While we await the development and availability of the next generation of influenza vaccines that need to elicit broad and durable protection, such as the desired “universal” influenza vaccine, continued improvements to existing and alternative platforms are an important public health priority.

## Electronic supplementary material

Appendix: Members of the Influenza Cell Culture Vaccine Working Group

## Data Availability

All the data used to conceive and write this manuscript are in the public domain.

## Electronic supplementary material

**Supplementary information** accompanies the paper on the *npj Vaccines* website (10.1038/s41541-018-0079-z).
