# Supplementary material for: Cell culture-derived influenza vaccines in the severe 2017–2018 epidemic season: a step towards improved influenza vaccine effectiveness
Source: NPJ Vaccines. 2018 Oct 9;3:44. doi: 10.1038/s41541-018-0079-z (PMC6177469; doi:10.1038/s41541-018-0079-z)
Supplement: Supplementary file 1 — Appendix: Members of the Influenza Cell Culture Vaccine Working Group [file 41541_2018_79_MOESM1_ESM.docx]

**APPENDIX**

**Members of The Influenza Cell Culture Vaccine Working Group and their affiliations at the time the work was conducted ^[[1]](#footnote-1)^**

WHO Influenza Collaborating Center in Melbourne

Ian Barr

Joelle Dharmakumara

Heidi Peck

Scott Reddiex

Cleve Rynehart

Rob Shaw

Sally Soppe

WHO Influenza Collaborating Center in Atlanta

Ginger Atteberry

John Barnes

Li-Mei Chen

Ruben O. Donis

Vivien Dugan

Angie Foust

Rebecca Garten

Adam Johnson

Jacqueline Katz

Yumiko Matsuoka

Thomas Rowe

David Wentworth

Xiyan Xu

Bin Zhou

Thedi Ziegler

WHO Influenza Collaborating Center in London

Rod Daniels

Yipu Lin

John W McCauley

WHO Influenza Collaborating Center in Tokyo

Eri Nobusawa

Takato Odagiri

WHO Influenza Essential Regulatory Laboratory at the National Institutes of Biological Standards and Controls, London, UK

Othmar Engelhart

Philip Minor

James Robertson

Biomedical Advanced Development Authority (BARDA)

Michael Angelastro

Rick Bright

Vittoria Cioce

Armen Donabedian

Ruben O. Donis

Joseph Figlio

Robert Huebner

Michael Perdue

Thomas Warf

Novartis Vaccines (now Seqirus)

Simone Blayer

Philip Dormitzer

John Fox

Chris Gully

Avishek Nandi

Jeffrey Pavlicek

Ethan Settembre

Beverly Taylor

Heidi Trusheim

Theodore Tsai

1. Arranged alphabetically and grouped by institutional affiliation [↑](#footnote-ref-1)
